# Vascular risk, gait, behavioral, and plasma indicators of VCID

**DOI:** 10.1002/alz.13540

**Published:** 2023-11-06

**Authors:** Sheelakumari Raghavan, Scott A. Przybelski, Timothy G. Lesnick, Angela J. Fought, Robert I. Reid, Robel K. Gebre, B. Gwen Windham, Alicia Algeciras‐Schimnich, Mary M. Machulda, Maria Vassilaki, David S. Knopman, Clifford R. Jack, Ronald C. Petersen, Jonathan Graff‐Radford, Prashanthi Vemuri

**Affiliations:** ^1^ Department of Radiology Mayo Clinic Rochester Minnesota USA; ^2^ Department of Quantitative Health Sciences Mayo Clinic Rochester Minnesota USA; ^3^ Department of Information Technology Mayo Clinic Rochester Minnesota USA; ^4^ Department of Medicine University of Mississippi Medical Center Jackson USA; ^5^ Department of Laboratory Medicine and Pathology Mayo Clinic Rochester Minnesota USA; ^6^ Department of Psychology Mayo Clinic Rochester Minnesota USA; ^7^ Department of Neurology Mayo Clinic Rochester Minnesota USA

**Keywords:** diffusion magnetic resonance imaging (MRI), vascular contributions to cognitive impairment and dementia, white matter, white matter hyperintensities

## Abstract

**INTRODUCTION:**

Cost‐effective screening tools for vascular contributions to cognitive impairment and dementia (VCID) has significant implications. We evaluated non‐imaging indicators of VCID using magnetic resonance imaging (MRI)‐measured white matter (WM) damage and hypothesized that these indicators differ based on age.

**METHODS:**

In 745 participants from the Mayo Clinic Study of Aging (≥50 years of age) with serial WM assessments from diffusion MRI and fluid‐attenuated inversion recovery (FLAIR)‐MRI, we examined associations between baseline non‐imaging indicators (demographics, vascular risk factors [VRFs], gait, behavioral, plasma glial fibrillary acidic protein [GFAP], and plasma neurofilament light chain [NfL]) and WM damage across three age tertiles.

**RESULTS:**

VRFs and gait were associated with diffusion changes even in low age strata. All measures (VRFs, gait, behavioral, plasma GFAP, plasma NfL) were associated with white matter hyperintensities (WMHs) but mainly in intermediate and high age strata.

**DISCUSSION:**

Non‐imaging indicators of VCID were related to WM damage and may aid in screening participants and assessing outcomes for VCID.

**Highlights:**

- Non‐imaging indicators of VCID can aid in prediction of MRI‐measured WM damage but their importance differed by age.
- Vascular risk and gait measures were associated with early VCID changes measured using diffusion MRI.
- Plasma markers explained variability in WMH across age strata.
- Most non‐imaging measures explained variability in WMH and vascular WM scores in intermediate and older age groups.
- The framework developed here can be used to evaluate new non‐imaging VCID indicators proposed in the future.

## 1 BACKGROUND

Vascular contributions to cognitive impairment and dementia (VCID) are associated with myriads of outcomes, including cognitive, gait, and behavioral changes in older adults.^1^, ^2^, ^3^ The etiology of VCID is multifactorial and measured primarily through brain changes from cerebral small vessel disease (SVD).^4^, ^5^ SVD‐related white matter (WM) damage measured using diffusion magnetic resonance imaging (dMRI) and fluid‐attenuated inversion recovery (FLAIR)‐magnetic resonance imaging (MRI) are commonly utilized VCID biomarkers.^6^, ^7^

Health policymakers suggest community‐based management and prevention strategies for VCID that include symptomatic treatment, management of risk factors, and other non‐pharmacological interventions in a cost‐effective manner. Of note, a major recommendation made at the Alzheimer's Disease‐Related Dementias (ADRD) 2022 summit was to develop inexpensive VCID biomarkers that can identify those at high risk of VCID and can aid in the enrollment of participants in SVD prevention clinical trials.^8^ More recently, the Framework for Clinical Trials in Cerebral Small Vessel Disease (FINESSE) put forth recommendations to design VCID clinical trials and suggested dMRI and white matter hyperintensities (WMH) as markers for patient selection and assessing treatment efficacy in phase 2 trials.^9^ Furthermore, diffusion tensor imaging (DTI) has been proposed as a sensitive marker to capture changes over time, so it could act as a reliable surrogate biomarker in SVD clinical trials.^10^, ^11^, ^12^ However, performing MRI on large populations for screening and identification of a subset of individuals at high risk of VCID may not be feasible. Further, screening electronic health records for VCID (based on imaging) is not possible because routine MRIs are not commonly available. Therefore, there is an urgent need to develop methods that can identify those at higher risk of VCID with available non‐imaging measures.

While age is the strongest risk factor, sex, cardiovascular and lifestyle factors, and chronic kidney disease (CKD)^2^, ^6^, ^13^ also contribute to the risk of VCID. However, screening participants based on these conditions alone does not identify those at higher risk of VCID due to significant biological heterogeneity. Participants with VCID often experience motor impairment (slower gait speed and increased falls)^14^ and behavioral problems such as anxiety, depression, and apathy.^2^, ^15^ Recently, the use of blood‐based biomarkers has been proposed across various trial designs.^9^, ^16^, ^17^ However, there are limited studies evaluating the utility of elevated neurofilament light chain (NfL), a non‐specific marker of neuro‐axonal injury,^17^, ^18^ and glial acidic protein (GFAP), a marker of astroglial pathology due to neuronal damage,^19^ in predicting poorer WM health in SVD. While several non‐imaging indicators for VCID are available and could be very useful for screening of populations for VCID, they have not been evaluated rigorously. We hypothesized that the inclusion of vascular risk factors (VRFs), gait, behavioral, and plasma markers would assist in explaining greater variability in SVD‐related WM damage than vascular risk measures alone (which are currently utilized for screening of VCID) in a population setting.

**FIGURE 1 alz13540-fig-0001:**
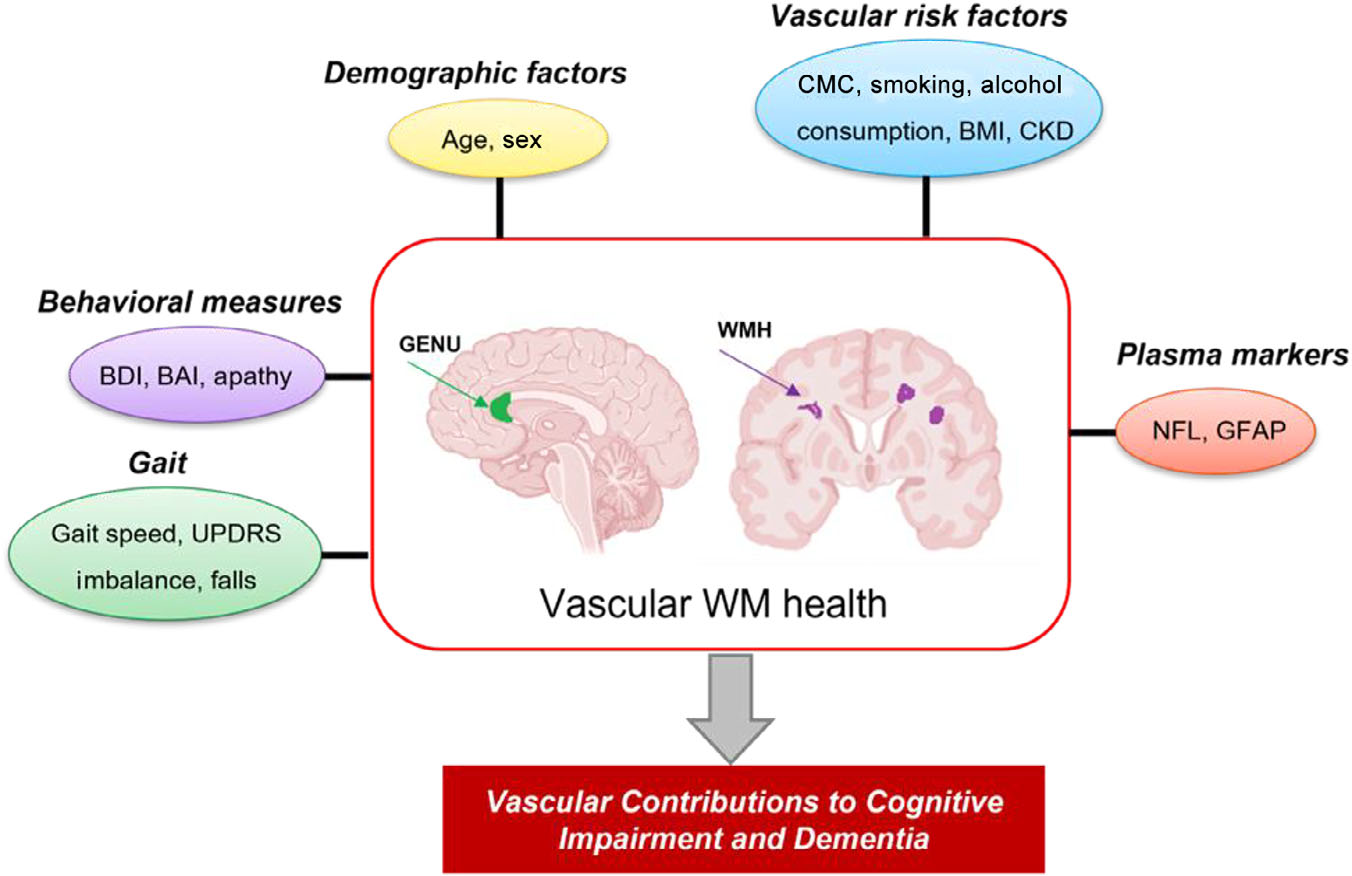
Conceptual model for measures evaluated to predict vascular contribution to cognitive impairment and dementia. WMH, white matter hyperintensity; CMC, cardiovascular and metabolic conditions; BMI, body mass index; CKD, chronic kidney disease; UPDRS, Unified Parkinson's disease rating scale; BDI, Beck depression inventory; BAI, Beck anxiety inventory; NfL, neurofilament light chain; GFAP, glial acidic protein.

Our primary goal was to compare non‐imaging indicators (VRFs, gait, behavioral, and plasma markers) of VCID based on their association with SVD‐related WM damage (Figure 1) across different age strata in a population‐based sample, while controlling for age and sex. Our secondary goal was to identify a comprehensive set of non‐imaging indicators of VCID that could aid in risk stratification and screening participants for clinical trials targeting those at high risk of VCID. Specifically, we focused on two VCID markers: WMH, a well‐established SVD biomarker, and fractional anisotropy of genu of corpus callosum (Genu‐FA), a regional dMRI marker that captures early systemic vascular injury.^20^, ^21^, ^22^ While the utilization of these two WM markers, Genu‐FA (reflecting early WM changes) and WMH (a biomarker that follows early diffusion changes), aids in evaluating the non‐imaging VCID indicators, we wanted to summarize the results using a composite metric that encompasses overall WM damage. Therefore, we used a combined Genu‐FA and WMH measure, proven to capture VCID effectively in the population.^23^

## 2 METHODS

### 2.1 Participants

The Mayo Clinic Study of Aging (MCSA) is an ongoing population‐based study of the residents of Olmsted County, Minnesota. MCSA utilizes the Rochester Epidemiology Project (REP) medical records‐linkage system to enumerate the Olmsted residents between the ages of 70 and 89 in 2004, with an age‐ and sex‐stratified design.^24^, ^25^ In 2012, the study was expanded to include participants aged 50 and older. The current study included 745 participants ≥50 years of age, who had at least two DTI and WMH measurements with a mean follow‐up of 3.2 years (0 to 7.8 years), and baseline demographic, VRFs, motor, behavioral, and plasma NFL and GFAP measures. The study design and diagnostic criteria for the MCSA population were described previously.^26^

As for standard protocol, registration, and patient consents, the study was approved by the Mayo Clinic and Olmsted Medical Center institutional review boards. Written informed consent was obtained from all participants.

### 2.2 Neuroimaging measures

#### 2.2.1 DTI markers

DTI sequence was processed and analyzed to obtain Genu‐FA using the method published previously (Appendix 1).^20^, ^27^ We considered Genu‐FA as an indicator of early WM damage due to systemic vascular injury because these tracts are disrupted in aging and SVD. We used the Johns Hopkins University or the JHU atlas to measure Genu‐FA from DTI scans.^28^

#### 2.2.2 White matter hyperintensities

A detailed description of the acquisition of 2D FLAIR images, segmentation, and WMH volume calculation was published previously.^29^ WMH volume scaled by total intracranial volume and log transformed due to skewness was used as the conventional SVD biomarker.

#### 2.2.3 Composite vascular WM score

Additionally, we utilized a previously validated principal component analysis^23^ to derive a vascular summary measure known as vascular WM score. We calculated the vascular WM score as the weighted sum of WMH and Genu‐FA, where a higher score indicates better WM health characterized by greater Genu‐FA and smaller WMH.

### 2.3 Measurement of predictor variables

#### 2.3.1 Demographic variables

We included self‐reported age, sex, and education measures ascertained from the baseline MCSA visit.

**RESEARCH IN CONTEXT**

1. **Systematic review**: We used PubMed and Google Scholar to review the literature on the association between non‐imaging indicators and SVD‐related WM damage as a marker of VCID. VRFs, gait, behavioral, and plasma markers significantly impacted microstructural and macrostructural WM integrity, but little is known about their utility for identifying elderly individuals with high vascular risk in stratified age groups.
2. **Interpretation**: Although the current set of indicators explained limited variability, the different sets of features accounted for variability in SVD‐related WM damage in each age stratum (<66.7, 66.7 to 76.5, >76.5 years). The VRFs and gait measures were associated with diffusion changes. While plasma NfL accounted for WMH variability in lower age strata, most non‐imaging measures explained variability in WMH in intermediate and high age groups.
3. **Future directions**: Future studies can help identify additional non‐imaging measures that can explain greater variability in SVD‐related WM damage which can be used as better screening tools for VCID.

#### 2.3.2 Vascular risk factors

Vascular health indicators of the participants were assessed from the health care records via trained nurses using the REP medical records‐linkage system. We included the presence of seven late‐life cardiovascular and metabolic conditions (hypertension, hyperlipidemia, cardiac arrhythmias, coronary artery disease, congestive heart failure, diabetes mellitus, and stroke) that were searched in a 5‐year capture frame and computed a composite score (referred to as cardiovascular and metabolic conditions [CMC]).^20^ We also included the presence of CKD and gathered the data on body mass index (BMI, weight in kilograms divided by height in meters squared), self‐reported smoking (never smoking vs former or currently smoking), and alcohol consumption from the MCSA visit.[Fig alz13540-fig-0001]

#### 2.3.3 Gait measures

Motor variables extracted from the REP included the recorded number of falls corresponding to gait and balance instability within 2 years preceding the MCSA visit. Gait speed (meters per second) was assessed with a GAITRite walkway over 7.62 m (25 feet) at the individual's usual pace. Unified Parkinson's disease rating scale (UPDRS) total score was ascertained during the MCSA clinical examination.

#### 2.3.4 Behavioral measures

Clinical anxiety and depression were assessed using Beck Anxiety Inventory (BAI)^30^ and Beck Depression Inventory‐II (BDI‐II),^31^ respectively. Each of these inventories is validated with 21 items that measure anxiety symptoms over the last week and depression symptoms over the past 2 weeks. A Likert scale ranging from 0 to 3, with a total score of 0 to 63, was used to rate the severity of each symptom, in which a cutoff score ≥10 in BAI indicated clinical anxiety and a cutoff score ≥13 in BDI‐II indicated clinical depression. Apathy was measured using neuropsychiatric inventory and indicated the presence of less interest in his or her usual activities and plans of others.

#### 2.3.5 Plasma measures

The detailed description of plasma collection, as well as NfL and GFAP measurements, were published previously.^32^, ^33^, ^34^, ^35^ The participant's blood was collected at a MCSA clinic visit after overnight fasting. Then the blood was centrifuged, aliquoted, and kept at −80°C. NfL and GFAP were measured on the Quanterix HD1 analyzer using the Simoa NF‐light or Simoa Neurology 4‐Plex E Advantage kit (N4PE, item no. 103670) per the manufacturer's instructions. After thawing and mixing, plasma samples were centrifuged for 5 min × 10,000 g. Samples were diluted at a 1:4 ratio using the instrument's onboard dilution protocol and run‐in duplicate from a single well each on a 96‐well plate. Eight‐point calibration curves and sample measurements were determined on the Simoa HD‐1 Analyzer software using a weighting factor 1/Y2 and a four‐parameter logistic curve‐fitting algorithm. Plasma NfL was measured using an in‐house digital ELISA on the Simoa‐HD1 Platform. Plasma GFAP was quantified using a biotinylated anti‐GFAP mouse monoclonal immunoglobulin (Ig)G antibody clone (capture antibody) and an anti‐GFAP rabbit IgG polyclonal antibody (detection antibody).^36^ The assay detects intact GFAP in addition to GFAP breakdown products (50 to 38 kDa).^36^ Two levels of quality control material were included, flanking the samples at the front and end of each batch. Both NFL and GFAP values were log‐transformed due to skewed distributions.

### 2.4 Statistical analysis

Participant characteristics were summarized with means and standard deviations (SDs) for the continuous variables and counts and percentages for the categorical variables. We categorized participants into three age groups based on tertiles to ensure an approximately equal number of participants in each stratum: low (<66.7 years), intermediate (66.7 to 76.5 years), and high (>76.5 years). This equal distribution of participants in tertiles facilitated the comparison of models across different age categories. The age groups allowed us to investigate potential differences in predictors, particularly in the lower age stratum (<66.7 years), where WMH accumulation is lower compared to Genu‐FA changes, which typically occur around the age of 50 years.^37^, ^38^ Further, the predictors that drive SVD changes in the older age strata may differ from those in the younger groups because SVD is highly prevalent in those >80 years of age.

#### 2.4.1 Evaluation of indicators of SVD WM damage

We fit separate linear mixed‐effects models with participant‐specific random intercepts to examine the indicators of longitudinal WM damage for all three measures (Genu‐FA, WMH, or vascular WM score) in three different age groups with age, sex, VRFs, gait measures, behavioral measures, and plasma markers as predictors. Here, we considered all main effects and interaction of all predictors with time. Therefore, we derived a total of 36 models, encompassing four models (VRFs, gait, behavioral, and plasma markers) per measure. The final parsimonious models were produced by a backward elimination respecting nested terms in which non‐significant terms were removed from the model one at a time. To produce parameter estimates with less than three decimal places, the outcome variables (FA, WMH, vascular WM score) were multiplied by 100. We report the parameter estimates, standard errors, and fixed‐effect *R* ^2^ (total for model and semi‐partial *R* ^2^ for main effect or combined main effect and interaction for each predictor, as appropriate) for each model.^39^

We also conducted sensitivity analyses to validate the suitability of CMC as a comprehensive summary measure of vascular and metabolic conditions. Here, we performed two sets of sensitivity analyses and compared their model performance to the CMC model: (1) by including midlife risk factors (hypertension, diabetes, and dyslipidemia) instead of CMC and (2) by including only five of the highly prevalent components of CMC (hypertension, diabetes, dyslipidemia, atrial fibrillation, and stroke instead of CMC). The performance of these sensitivity models, measured by the fixed‐effect *R* ^2^, ranged from 8% to 15%, comparable to the performance of CMC as a single measure. Based on these results, we selected CMC as the marker of vascular risk in all presented models.

#### 2.4.2 Building a composite non‐imaging VCID model for indicators of SVD‐related WM damage

After evaluating and comparing the individual indicators based on predictor models of the longitudinal WM damage (Genu‐FA, WMH, and vascular WM score), we wanted to identify the best minimal set of predictors. Therefore, we fit multivariable linear mixed‐effect models with vascular WM score as the outcome in each age strata. The base model consisting of baseline age, sex, VRFs, and their interaction were always included in all models. Using these base models, additional variables (gait, behavioral, and plasma markers) were added to test for their usefulness above and beyond the base model.

We avoided correction for multiple comparisons, which relies on a universal null hypothesis, to prevent inflating Type II error probabilities.^40^, ^41^

## 3 RESULTS

The overall participant characteristics are shown in Table 1. The mean (SD) age of overall participants was 71.4 (9.3) years, 55% were males, and 90% of the sample was cognitively unimpaired. Out of 745 participants, 246 were in the low‐age group, 249 were in the intermediate‐age group, and 250 were in the high‐age group.

**TABLE 1:** Characteristics table of all serial imaging participants with mean (SD) listed for continuous variables and count (%) for categorical variables.

Characteristics	All participants (*n* = 745)	Low (*n* = 246)	Intermediate (*n* = 249)	High (*n* = 250)
**Demographics**				
Age, years	71.4 (9.3)	60.6 (4.63)	72.2 (2.8)	81.4 (3.9)
Males, no. (%)	411 (55%)	130 (52.63%)	131 (52.61%)	150 (60%)
APOE4 carrier status, no. (%)	212 (28%)	68 (28%)	76 (31%)	68 (27%)
Cognitively unimpaired, no. (%)	669 (90%)	235 (95%)	230 (92%)	204 (82%)
Total follow‐up time points, no. (%)				
1	485 (65%)	208 (85%)	149 (60%)	128 (51%)
2	195 (26%)	36 (15%)	80 (32%)	79 (32%)
3	57 (77%)	2 (8%)	19 (76%)	36 (14%)
4+	8 (1%)	–	1 (4%)	7 (3%)
**Intellectual enrichment**				
Education, years	14.73 (2.7)	15.11 (2.41)	14.56 (2.63)	14.52 (2.93)
**Cognitive measures**				
Global z‐score	0.08 (1.08)	0.66 (0.94)	0.02 (0.92)	−0.43 (1.10)
Attention z‐score	−0.10 (1.08)	0.51 (0.90)	0.02 (0.90)	−0.56 (1.17)
**Vascular risk factors**				
CMC	1.98 (1.3)	1.43 (1.02)	1.99 (1.25)	2.52 (1.26)
Smoking, no. (%)	320 (42.9%)	104 (42.1%)	115 (46.2%)	101 (40.4%)
Alcohol consumption, no. (%)	35 (5%)	16 (7%)	7 (3%)	12 (5%)
BMI (kg/m^2^)	28.16 (4.9)	29.29 (5.3)	28.52 (4.8)	26.70 (4.2)
CKD, no. (%)	50 (7%)	7 (2%)	15 (6%)	28 (11%)
**Motor measures**				
Gait speed (m/s)	1.18 (0.48)	1.34 (0.65)	1.17 (0.43)	1.04 (0.21)
Falls, no. (%)	19 (3%)	2 (8%)	6 (2%)	11 (4%)
UPDRS	1.11(3.0)	0.562 (2.1)	0.807 (2.5)	1.95 (4.0)
**Behavioral measures**				
BAI	2.74 (3.8)	3.09 (4.2)	2.18 (3.0)	2.97 (4.1)
BDI	4.61 (4.9)	4.90 (5.6)	4.08 (4.1)	4.86 (4.9)
Apathy, no. (%)	28 (4%)	6 (2%)	9 (4%)	13 (5%)
**Plasma markers**				
NfL (pg/mL)	27.37 (20.22)	17.22 (12.47)	24.45 (12.95)	40.30 (24.99)
GFAP (pg/mL)	108.10 (61.25)	69.63 (34.23)	102.67 (42.28)	151.52 (69.96)
**Imaging markers**				
Genu‐FA	0.60 (0.05)	0.62 (0.04)	0.60 (0.04)	0.57 (0.05)
WMH/TIV (%)	0.88 (1.10)	0.44 (0.70)	0.82 (0.86)	1.38 (1.15)
Vascular WM Score	0.84 (0.67)	1.30 (0.59)	0.81 (0.58)	0.40 (0.52)

Abbreviations: Genu‐FA, fractional anisotropy of genu of corpus callosum; WMH, white matter hyperintensity; CMC, cardiovascular and metabolic conditions; BMI, body mass index; CKD, chronic kidney disease; UPDRS, Unified Parkinson's disease rating scale; BAI, Beck anxiety Inventory; BDI, Beck depression inventory; NfL, neurofilament light chain; GFAP, glial fibrillary acidic protein.

### 3.1 Comparison between non‐imaging indicators of WM damage

**FIGURE 2:**
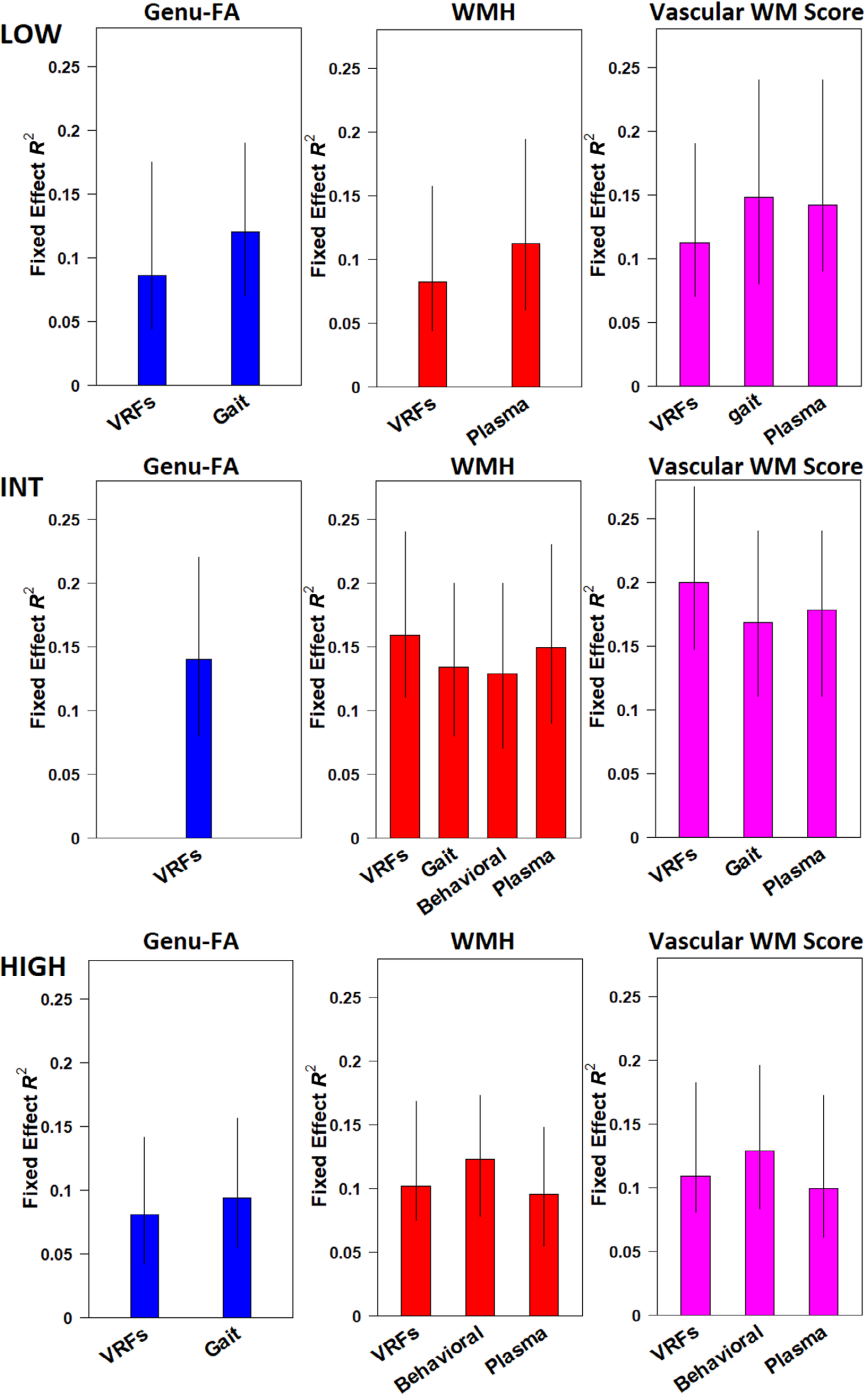
Independent contributions of significant group measures to white matter damage in low‐, intermediate‐ (INT), and high‐age strata. Shown is the model performance using fixed‐effect *R* ^2^ based on significant predictors across age groups. Genu‐FA, fractional anisotropy of genu of corpus callosum; WM, white matter; WMH, white matter hyperintensity; VRF, vascular risk factor.

The models evaluating the independent associations between predictors of interests with longitudinal Genu‐FA, WMH, and vascular WM scores as outcomes in three different age strata are shown in Figure 2 (total fixed‐effect *R* ^2^) and Supplementary Table 1. Supplemental Figure 1 illustrates the semi‐partial *R* ^2^ for each significant indicator.

#### 3.1.1 Indicators of Genu‐FA

VRFs as well as gait measures explained some of the variability (total fixed‐effect *R* ^2^) in Genu‐FA in both low (8.6% and 12.1%, respectively) and high‐age strata (8.1% and 9.4%, respectively), while VRFs alone explained some of the variability in Genu‐FA in the intermediate‐age strata (14%). However, no significant contribution was observed from behavioral or plasma measures in the Genu‐FA model performance. Across all Genu‐FA models (shown in Supplementary Table 1), older age was associated with lower microstructural WM integrity, whereas being male was associated with better WM microstructure. Smoking status was only associated with lower Genu‐FA in the low‐age strata (β = −1.07, *p =* .026). While high BMI was associated with lower Genu‐FA in intermediate‐age strata (β = −0.18, *p =* .001), high CMC was associated with lower baseline Genu‐FA in high‐age strata (β = −0.54, *p =* .036). Higher UPDRS scores at baseline were associated with lower Genu‐FA in low‐ and high‐age strata (β = −0.31, *p =* .008 and β = −0.22, *p =* .006, respectively). Additionally, in the low‐age strata, a slower baseline gait speed and history of falls predicted a greater rate of decline in Genu‐FA longitudinally (β = 0.39, *p =* .022 and β = 1.41, *p =* .007, respectively).

#### 3.1.2 Indicators of WMH

VRFs and plasma markers explained some of the WMH variability in the low‐age strata (total fixed effect *R* ^2^ = 8.2% and 11.2%, respectively), whereas in the intermediate‐ and high‐age groups, gait and behavioral measures additionally explained some of the variance in WMH (as shown in Figure 2 and Supplemental Figure 1). In all WMH models in the low‐ and high‐age groups, older age was associated with greater baseline WMH, while a faster rate of increase of WMH burden was observed only in the intermediate‐age strata (*p* < .05). Male sex was related to lower WMH in the intermediate‐ and high‐age strata models, except gait and plasma models in the intermediate‐ and high‐age strata, respectively. Interestingly, as shown in Supplemental Table 1, higher baseline CMC was associated with faster rate of WMH accumulation in the intermediate‐age strata (β = 0.71, *p =* .019) and slower rate of accumulation in the high‐age age groups (β = −0.56, *p =* .021). CKD was associated with a faster rate of accumulation of WMH in the low‐age groups (β = 9.11, *p =* .026), whereas smoking status exhibited a similar greater rate of increase of WMH in the high‐age stratum (β = 1.48, *p =* .01). History of falls at baseline were related to higher WMH in the intermediate‐age strata (β = 95.49, *p =* .004). Higher baseline BAI was associated with a greater WMH burden in the high‐age strata (β = 3.76, *p =* < .001), while a higher baseline BDI was associated with a greater rate of increase in WMH burden longitudinally in the intermediate‐age groups (β = 0.19, *p =* .044). Additionally, higher baseline plasma NfL levels were associated with greater WMH in both low‐age strata (β = 42.0, *p =* .004), while higher baseline plasma GFAP values were associated with greater WMH in the intermediate‐age strata (β = 35.80, *p =* .008). Furthermore, NfL was associated with a faster rate of increase in WMH in the high‐age strata (β = 1.84, *p =* .005).

#### 3.1.3 Indicators of vascular WM score

While VRFs, gait, and plasma markers accounted for some of the variability in the vascular WM score in the low‐age strata (total fixed effect *R* ^2^ = 11.2, 14.8, and 14.2%, respectively), there was an additional contribution from behavioral measures in the intermediate‐ and high‐age strata (Figure 2 and Supplemental Table 1). In all vascular WM score models, older age was associated with lower WM integrity and had a greater age‐related decline only in the intermediate‐age stratum. Notably, higher baseline CMC was associated with a faster rate of decline in vascular WM scores in the intermediate‐age stratum (β =  −0.55, *p =* .007) and a slower decline in the high‐age stratum (β = 0.37, *p =* .021). Baseline CKD was associated with greater rate of WM decline in low‐age groups (β = −5.71, *p =* .034), whereas smoking was related to greater decline in the high‐age groups (β = −1.01, *p =* .009). Additionally, greater UPDRS scores were associated with lower baseline vascular WM score in the low‐age stratum (β = −3.95, *p =* .007), whereas history of falls at baseline were associated with a lower baseline vascular WM score in the intermediate‐age stratum (β = −64.05, *p =* .003). Higher baseline BAI was associated with lower baseline vascular WM score in the high‐age strata (β = −2.60, *p =* < .001). Higher baseline plasma NfL levels were associated with lower vascular WM scores in the low‐age stratum (β = −21.81, *p =* .003), whereas higher baseline GFAP values were associated with lower vascular WM scores in the intermediate‐age stratum (β = −22.51, *p =* .008). In addition, a higher baseline NfL predicted a faster rate of decline in the vascular WM score in the high‐age stratum (β = −1.29, *p =* .003).

### 3.2 Composite non‐imaging VCID model

The findings of the two mixed‐effect models: (i) base model with VRFs after adjusting for age and sex and (ii) final composite parsimonious model derived by adding gait, behavioral, and plasma markers on top of base model with vascular WM score as the outcome are shown in Table 2. The composite model (with all individual sets of predictors on top of the base model) improved the model prediction modestly in all age groups (fixed‐effect model *R* ^2^ increased from 11.2% to 16.5% in the low‐age group, 20% to 23.4% in the intermediate‐age group, and 10.9% to 15.2% in the high‐age group). While older age was associated with lower WM integrity (*p* < .005) in all age groups, male sex was related to better WM health in the intermediate‐ and high‐age strata (*p* < .05) in the composite models. Interestingly, in the low‐age group, higher UPDRS and elevated plasma NfL levels were associated with lower baseline vascular WM scores (β = −3.79, *p =* .009 and β = −21.87, *p =* .003, respectively). Higher CKD frequency was associated with a greater rate of decline in WM health (β = −5.76, *p =* .003). In the intermediate‐age group, history of falls and plasma GFAP levels were linked to poorer baseline WM health. Furthermore, higher CMC was associated with a greater rate of decline in the vascular WM score (β = −0.62, *p =* .002), whereas higher UPDRS was associated with a slower rate of decline in the intermediate‐age stratum (β = 0.27, *p =* .014). In the high‐age group, worse BAI was associated with poorer baseline WM health. While higher plasma NfL levels and smoking status were associated with a greater rate of WM decline in the high‐age group (β = −1.37, *p =* .001 and β = −1.03, *p =* .007), higher CMC was associated with a slower rate of decline in the vascular WM score (β = 0.41, *p =* .012).

**TABLE 2:** Linear mixed‐effect models evaluating the contribution of gait, behavioral, and plasma measures on top of base model with vascular risk factors to predict white matter damage (vascular WM score) after adjusting for age and sex across age groups.

		Low		Intermediate		High	
Models	Predictor	Estimate (SE)	*p* value	Estimate (SE)	*p* value	Estimate (SE)	*p* value
Base model	Intercept	351.65 (40.33)	**<.001**	493.86 (83.66)	**<.001**	279.45 (66.12)	**<.001**
	**Time**	−1.49 (0.38)	**<.001**	−3.87 (0.47)	**<.001**	−5.41 (0.44)	**<.001**
	**Age**	−3.73 (0.66)	**<.001**	−5.63 (1.16)	**<.001**	−2.90 (0.82)	**<.001**
	**Male**	–	–	18.02 (6.46)	**.006**	14.96 (6.56)	**.023**
	**CMC**	–	–	−8.70 (2.60)	**<.001**	−5.66 (2.57)	**.029**
	**Smoking**	–	–	–	–	−1.27 (6.15)	.84
	**CKD**	1.47 (18.79)	.94	–	–	–	–
	**Time×CMC**	–	–	−0.55 (0.20)	**.007**	0.37 (0.16)	**.021**
	**Time×Smoking**	–	–	–	–	−1.01 (0.39)	**.009**
	**Time×CKD**	−5.71 (2.68)	**.034**	–	–	–	–
**R^2^ (total fixed effect)**		**0.112 (0.07 to 0.189)**		**0.201 (0.147 to 0.275)**		**0.109 (0.08 to 0.182)**	
Base model + Gait measures + Behavioral measures+ Plasma markers	Intercept	404.27 (44.60)	**<.001**	471.90 (83.70)	**<.001**	303.82 (66.71)	**<.001**
	**Time**	−1.38 (0.38)	**<.001**	−3.93 (0.46)	**<.001**	1.22 (2.12)	.57
	**Age**	−3.05 (0.67)	**<.001**	−4.45 (1.25)	**<.001**	−2.46 (0.83)	**.003**
	**Male**	–	–	15.85 (6.50)	**.015**	13.68 (6.43)	**.034**
	**CMC**	–	–	−7.70 (2.60)	**.003**	−4.49 (2.54)	.079
	**Smoking**	–	–	–	–	0.69 (6.04)	.91
	**CKD**	0.85 (18.28)	.96	–	–	–	–
	UPDRS	−3.79 (1.44)	**.009**	−1.40 (1.31)	.29	–	–
	Falls	–	–	−41.58 (21.02)	**.049**	–	–
	BAI	–	–	–	–	−2.45 (0.77)	**.002**
	NfL	−21.87 (7.27)	**.003**	–	–	−11.21 (6.93)	.11
	GFAP	–	–	−20.15 (8.29)	**.016**	–	–
	**Time×CMC**	–	–	−0.62 (0.2)	**.002**	0.41 (0.16)	**.012**
	**Time×Smoking**	–	–	–	–	−1.03 (0.38)	**.007**
	**Time×CKD**	−5.76 (2.68)	**.003**	–	–	–	–
	Time**×**UPDRS	–	–	0.27 (0.11)	**.014**	–	–
	Time**×**NfL	–	–	–	–	−1.37 (0.43)	**.001**
**R^2^ (total fixed effect)**		**0.165 (0.125 to 0.207)**		**0.234 (0.18 to 0.309)**		**0.152 (0.114 to 0.224)**	

*Note*: We have shown only significant predictors and indicated base variables in bold.

Abbreviations: CMC, cardiovascular and metabolic conditions; CKD, chronic kidney disease; UPDRS, Unified Parkinson's disease rating scale; BAI, Beck anxiety inventory, NfL, neurofilament light chain; GFAP, glial acidic protein.

## 4 DISCUSSION

While MRI measures are the standardized methods to capture underlying VCID disease processes, several non‐imaging indicators can also inform us on the extent of SVD‐related brain damage and may be useful for screening of large populations for VCID. This prospective cohort study has comprehensively investigated non‐imaging indicators in relation to longitudinal WM changes across three age strata. Our two important conclusions were as follows: (1) VRFs and gait changes were associated with variability in Genu‐FA, which is reflective of early SVD in all age strata. VRFs, gait, behavioral, and plasma measures were associated with WMH and vascular WM score particularly in the intermediate and higher tertiles. (2) Although the contribution is moderate, the composite model with all non‐imaging measures (VRFs, gait, behavioral, plasma markers) improved model performance in predicting vascular WM scores over base model with VRFs alone. The framework proposed here can be utilized for the discovery of newer and more effective non‐imaging indicators of VCID.

### 4.1 Vascular health and WM damage

Poor vascular health impacts WM health,^42^ with the frontal lobe WM being particularly susceptible to vascular lesions^43^, ^44^ and microstructural WM damage.^7^, ^42^ In the independent models, we found the deleterious effects of smoking on Genu‐FA in low‐age group, highlighting the utility of measuring WM microstructure in midlife. We also observed a significant association between BMI and CMC with Genu‐FA in the intermediate‐ and high‐age groups, respectively. Together, these findings extend past evidence from us^20^, ^21^, ^37^ and others,^12^, ^45^ suggesting an association between modifiable risk factors (hypertension, diabetes, hyperlipidemia, obesity, and smoking status) and reduced WM integrity of the genu of the corpus callosum before overt WMH.^37^, ^46^, ^47^ However, we observed no association between VRFs and baseline WMH in the low‐age strat stratum, as WMH changes tend to occur mostly after 65 years of age. Our results provide evidence that early detection and treatment of those at higher risk of SVD in younger ages (<70 years) before substantial WMH changes is crucial to preserve WM integrity and mitigate the risk of VCID. Additionally, CKD predicted the rate of increase in WMH in the low‐age stratum. Nevertheless, in the intermediate‐age group, our findings are consistent with previous studies, which showed greater baseline and progression of WMH in association with VRFs.^37^, ^38^, ^48^, ^49^ It might be attributed to underlying mechanisms such as vascular risk‐induced hypoperfusion, altered cerebrovascular reactivity, and increased blood–brain barrier permeability.^50^ Furthermore, we found slower progression of WMH in association with CMC at the eighth decade of life, which is consistent with previous findings.^48^

Additionally, in the independent and final composite models, we found associations between VRFs and vascular WM score across all age tertiles, supporting the usefulness of the composite measure, which is reflective of both early and late WM changes due to systemic vascular injury. The overall contribution of VRFs to WM damage was less pronounced in the high‐age group compared to intermediate group, likely due to the greater prevalence of risk factors coupled with greater WM damage in the older age.

### 4.2 Motor impairment and WM damage

Motor impairment (impaired gait speed and falls) is considered one of the main indicators of functional disability in VCID, and a recent review suggested the interrelationship between gait abnormalities and SVD brain changes as key markers to distinguish SVD individuals from dementia in the older population.^51^ We found that slower gait speed and presence of falls were associated with decline in the integrity of genu in low‐age groups, corroborating previous findings.^52^, ^53^ Additionally, we found lower Genu‐FA in association with UPDRS in both younger and older age groups, consistent with the Rotterdam study that reported the most significant changes in the genu of corpus callosum in vascular parkinsonism participants compared to those without.^54^ In fact, the corpus callosum and its projected motor pathways are responsible for higher‐order motor functions^55^, ^56^ (planning, initiation, execution, and control) and further support the idea that impairment of motor function in aging might be due to a “disconnection syndrome” and can be used as an early marker of SVD disease progression.

We also observed that in intermediate groups, falls were associated with WMH and vascular WM score in independent models. These results are in line with a recent meta‐analysis that showed associations between gait or falls and SVD burden (including WMH and microbleeds).^14^ Interestingly, UPDRS scores were associated with vascular WM score in younger age groups in the composite models. We also observed a slower rate of decline in the composite measure in association with UPDRS in the intermediate groups, which is likely reflective of correlated variables in model (Table 2). Together, our findings emphasize the need to account for functional disability when assessing the progression of WM damage in older adults. Further research is needed to explore the neurobiological basis of gait and WM decline.

### 4.3 Behavioral measures and WM damage

Neuropsychiatric symptoms, such as anxiety, depression, and apathy, are the common clinical presentations of VCID,^2^, ^15^ and evidence also supports the correlation between behavioral manifestations and imaging markers of SVD.^15^, ^57^, ^58^ We found an association between higher anxiety scores and higher WMH, as well as lower vascular WM score within the high‐age stratum, as evidenced in both univariate and multivariate models. As expected, baseline depression scores predicted greater progression of WMH in the intermediate‐age group due to a greater incidence of WMH and depression. Together these findings support and extend the previous findings.^59^ However, there was no contribution from behavioral symptoms to baseline Genu‐FA, which may indicate that behavioral symptoms are associated with a greater extent of WM damage (as captured by WMH). Of note, the relationship between anxiety and SVD is understudied, but our study supports the notion that future investigations would bolster these observations.

### 4.4 Blood biomarkers and WM damage

NfL and GFAP have been proposed as promising biomarkers for VCID in addition to MRI,^60^ but their utility in identifying high‐risk individuals remains elusive. While both markers showed associations with WM damage, the results were not straightforward. When we tested them simultaneously in univariate and composite models, NfL was associated with baseline WMH and vascular WM score in low‐ and high‐age groups^18^, ^61^, ^62^; however, GFAP was associated with WMH ^63^ and vascular WM score in the intermediate groups, indicating the usefulness of plasma markers in capturing WM damage. Consistent with previous findings,^11^, ^61^, ^62^ NfL predicted the progression of WMH in low‐ and high‐age groups, suggesting it as a more valuable prognostic biomarker for SVD progression. Because NFL and GFAP are non‐specific markers of neuronal injury, further work needs to be done to tease out their utility of VCID in the presence of other dementia etiologies. The associations of WM damage with GFAP levels in the intermediate tertile was surprising. The elevated GFAP and NfL levels in the intermediate tertile likely represent individuals at higher risk of progressing to MCI/dementia due to elevated AD and VCID pathologies. The high‐age tertile likely has survivor bias and may not have the same associations as the intermediate‐age group.

### 4.5 The usefulness of non‐imaging measures for VCID

To screen large populations for VCID, obtaining high‐quality MRI for surrogate measures like DTI and quantitative WMH is challenging, as this information is not routinely available during annual physician visits. Current efforts, such as MarkVCID^64^ and lifestyle intervention studies,^65^ focus on screening high‐risk individuals based on VRFs, but there is significant heterogeneity in WM damage, even among those at high risk. As suggested by Hulleck et al.,^66^ quantitative gait analysis remains largely associated with research institutions and not well leveraged in clinical settings. However, there is increasing evidence that incorporating gait assessments and recording easily accessible gait measures as part of routine practice may be extremely helpful in the assessment of health outcomes,^67^, ^68^, ^69^ particularly in our context for identifying VCID. Additionally, as plasma measures become more widely available during clinical visits for the elderly, incorporating gait and plasma markers with VRFs may help identify those with worse vascular WM scores.

When we undertook these analyses based on the key recommendations of ADRD Summit 2022 for VCID (development of cost‐effective markers for identifying individuals at risk of VCID to facilitate participant enrollment in SVD prevention trials), we did not expect a low variability of the non‐imaging indicators in predicting WM changes. However, these detailed analyses shed light on the differences in the predictive power of the markers by age in predicting early versus late WM changes. Future research will help identify additional relevant measures (education, marital status, area deprivation index, genetics, medication status, diet, quantitative measures of gait, and inflammatory markers) using the framework proposed here to improve sensitivity in screening for VCID.

The strengths of this study include the community‐based population with reasonable sample size, availability of concurrent plasma NfL and GFAP, serial neuroimaging markers of SVD (mean follow‐up of 3.2 years), clinical, and multiple gait/behavior assessments. Limitations include the narrow focus on SVD‐related WM changes (not microbleeds and infarcts), the use of a composite score for VRFs without accounting for medication status (dose and duration), and the limited number of gait and behavioral variables. Further, the causal links between the non‐imaging indicators and imaging measures were not evaluated but will be part of future studies.

## 5 CONCLUSION

Non‐imaging indicators can be useful for screening and early identification of individuals at high risk of VCID in large populations. We successfully utilized MRI‐based WM changes to screen for non‐imaging indicators of VCID and found differences in these indicators across different age strata. Our results support the inclusion of VRFs, gait, and plasma markers for explaining variability in SVD‐related WM changes compared to VRFs alone. Future studies should focus on the development and discovery of better measures that can explain greater variability than is shown here.

## CONFLICTS OF INTEREST STATEMENT

The authors do not have any pertinent disclosures relevant to this study. Author disclosures are available in the supporting information.

## CONSENT STATEMENT

All participants provided informed consent.

## Supporting information

Supporting Information

Supporting Information
