# Supplementary material for: Vascular risk, gait, behavioral, and plasma indicators of VCID
Source: Alzheimers Dement. 2023 Nov 6;20(2):1201–13. doi: 10.1002/alz.13540 (PMC10916988; doi:10.1002/alz.13540)
Supplement: Supplementary file 1 — Supporting Information [file ALZ-20-1201-s001.docx]

**Supplemental Materials**

| **Supplemental Table 1:** Mixed effect models evaluating the indicators to white matter damage in three different age groups. We have shown only significant predictors. | | | | | | | |
| --- | --- | --- | --- | --- | --- | --- | --- |
|  |  | **LOW** | | **INTERMEDIATE** | | **HIGH** | |
| Models | Predictor | Estimate (S.E) | P-value | Estimate (S.E) | P-value | Estimate (S.E) | P-  value |
| **Models with Genu-FA as an outcome** | | | | | | | |
| Vascular risk factors | Intercept | 70.23 (3.10) | **<0.001** | 89.92 (7.03) | **<0.001** | 76.33 (6.67) | **<0.001** |
|  | Time | -0.19 (0.04) | **<0.001** | -0.45 (0.03) | **<0.001** | -0.40 (0.03) | **<0.001** |
|  | Age | -0.14 (0.05) | **0.007** | -0.35 (0.09) | **<0.001** | -0.23 (0.08) | **0.006** |
|  | Male | 1.53 (0.47) | **0.001** | 1.25 (0.50) | **0.013** | 1.50 (0.65) | **0.022** |
|  | CMC | - | - | - | - | -0.54 (0.26) | **0.036** |
|  | Smoking | -1.07 (0.48) | **0.026** | - | - | - | - |
|  | BMI | - | - | -0.18 (0.06) | **0.001** | - | - |
| **R^2^ (total fixed effect)** |  | **0.086 (0.044-0.175)** |  | **0.14 (0.08-0.22)** |  | **0.081 (0.042-0.141)** |  |
| Gait measures | Intercept | 66.85 (3.44) | **<0.001** | 81.89 (6.70) | **<0.001** | 74.68 (6.67) | **<0.001** |
|  | Time | -0.68 (0.21) | **0.002** | -0.45 (0.03) | **<0.001** | -0.40 (0.03) | **<0.001** |
|  | Age | -0.12 (0.05) | **0.015** | -0.31 (0.09) | **<0.001** | -0.22 (0.08) | **0.007** |
|  | Male | 1.40 (0.47) | **0.003** | 1.25 (0.51) | **0.016** | 1.51 (0.65) | **0.021** |
|  | Gait speed | 1.84 (1.06) | 0.09 | - | - | - | - |
|  | UPDRS | -0.31 (0.11) | **0.008** | - | - | -0.22 (0.08) | **0.006** |
|  | Falls | -2.31 (2.70) | 0.39 | - | - | - | - |
|  | Time*Gait speed | 0.39 (0.17) | **0.022** | - | - | - | - |
|  | Time*Falls | 1.41 (0.52) | **0.007** | - | - | - | - |
| **R^2^ (total fixed effect)** |  | **0.121 (0.068-0.194)** |  | **0.11 (0.069-0.182)** |  | **0.094 (0.055-0.156)** |  |
| Behavioral measures | Intercept | 70.60 (3.13) | **<0.001** | 81.89 (6.70) | **<0.001** | 77.24 (6.70) | **<0.001** |
|  | Time | -0.18 (0.04) | **<0.001** | -0.45 (0.03) | **<0.001** | -0.40 (0.03) | **<0.001** |
|  | Age | -0.15 (0.05) | **0.004** | -0.31 (0.09) | **<0.001** | -0.26 (0.08) | **0.002** |
|  | Male | 1.50 (0.48) | **0.002** | 1.25 (0.51) | **0.016** | 1.38 (0.65) | **0.036** |
| **R^2^ (total fixed effect)** |  | **0.07 (0.044-0.105)** |  | **0.11 (0.069-0.182)** |  | **0.068 (0.035-0.117)** |  |
| Plasma markers | Intercept | 70.60 (3.13) | **<0.001** | 81.89 (6.70) | **<0.001** | 77.24 (6.70) | **<0.001** |
|  | Time | -0.18 (0.04) | **<0.001** | -0.45 (0.03) | **<0.001** | -0.40 (0.03) | **<0.001** |
|  | Age | -0.15 (0.05) | **0.004** | -0.31 (0.09) | **<0.001** | -0.26 (0.08) | **0.002** |
|  | Male | 1.50 (0.48) | **0.002** | 1.25 (0.51) | **0.016** | 1.38 (0.65) | **0.036** |
| **R^2^ (total fixed effect)** |  | **0.07 (0.044-0.105)** |  | **0.11 (0.069-0.182)** |  | **0.068 (0.035-0.117)** |  |
| **Models with WMH as an outcome** | | | | | | | |
| Vascular risk factors | Intercept | -941.88 (79.0) | **<0.001** | -1018.25(133.8) | **<0.001** | -796.32 (96.10) | **<0.001** |
|  | Time | 1.84 (0.57) | **0.001** | -16.21 (10.58) | **<0.001** | 7.75 (0.70) | **<0.001** |
|  | Age | 6.10 (1.3) | **<0.001** | 6.93 (1.85) | **<0.001** | 4.10 (1.20) | **<0.001** |
|  | Male | - | - | -28.65 (10.25) | **0.006** | -20.93 (9.62) | **0.031** |
|  | CMC | - | - | 11.24 (4.12) | **0.007** | 7.96 (3.80) | **0.04** |
|  | Smoking | - | - | - | - | 1.90 (9.03) | 0.84 |
|  | CKD | -12.05 (36.51) | 0.74 | - | - | - | - |
|  | Time*Age | - | - | 0.30 (0.15) | **0.041** | - | - |
|  | Time*CMC | - | - | 0.71 (0.30) | **0.019** | -0.56 (0.24) | **0.021** |
|  | Time*Smoking | - | - | - | - | 1.48 (0.59) | **0.01** |
|  | Time*CKD | 9.11 (4.06) | **0.026** | - | - | - | - |
| **R^2^ (total fixed effect)** |  | **0.082 (0.044-0.157)** |  | **0.159 (0.11-0.24)** |  | **0.102 (0.075-0.168)** |  |
| Gait measures | Intercept | -942.28 (78.80) | **<0.001** | -1060.51 (133.8) | **<0.001** | -800.92 (96.78) | **<0.001** |
|  | Time | 2.02 (0.57) | **<0.001** | -18.34 (10.62) | 0.085 | 6.95 (0.31) | **<0.001** |
|  | Age | 6.10 (1.30) | **<0.001** | 7.58 (1.85) | **0.004** | 4.40 (1.20) | **<0.001** |
|  | Male | - | - | - | - | -18.75 (9.45) | **0.048** |
|  | Falls | - | - | 95.49 (33.24) | **0.004** | - | - |
|  | Time*Age | - | - | 0.35 (0.15) | **0.018** | - | - |
| **R^2^ (total fixed effect)** |  | **0.082 (0.044-0.158)** |  | **0.134 (0.08-0.20)** |  | **0.091 (0.055-0.157)** |  |
| Behavioral measures | Intercept | -942.28 (78.78) | **<0.001** | -1028.54 (136.27) | **<0.001** | -806.78 (94.84) | **<0.001** |
|  | Time | 2.02 (0.57) | **<0.001** | -19.32 (10.58) | 0.07 | 6.96 (0.31) | **<0.001** |
|  | Age | 6.10 (1.30) | **<0.001** | 7.43 (1.87) | **<0.001** | 4.34 (1.20) | **<0.001** |
|  | Male | - | - | -24.55 (10.32) | **0.018** | -19.09 (9.30) | **0.04** |
|  | BDI | - | - | -1.43 (1.27) | 0.26 | - | - |
|  | BAI | - | - | - | - | 3.76 (1.12) | **<0.001** |
|  | Time*Age | - | - | 0.35 (0.15) | **0.016** | - | - |
|  | Time*BDI | - | - | 0.19 (0.09) | **0.044** | - | - |
| **R^2^ (total fixed effect)** |  | **0.082 (0.035-0.158)** |  | **0.129 (0.07-0.20)** |  | **0.123 (0.078-0.173)** |  |
| Plasma markers | Intercept | -1062.03 (87.74) | **<0.001** | -999.26 (134.9) | **<0.001** | -832.43 (99.94) | **<0.001** |
|  | Time | 2.02 (0.57) | **<0.001** | -18.26 (10.62) | 0.09 | -2.07 (3.23) | 0.52 |
|  | Age | 5.19 (1.31) | **<0.001** | 5.41 (2.01) | **0.008** | 3.60 (1.23) | **0.004** |
|  | Male | - | - | -23.67 (10.16) | **0.021** | - | - |
|  | NfL | 42.0 (14.37) | **0.004** | - | - | 17.34 (10.41) | 0.09 |
|  | GFAP | - | - | 35.80 (13.30) | **0.008** | - | - |
|  | Time*Age | - | - | 0.35 (0.15) | **0.018** | - | - |
|  | Time*NfL | - | - | - | - | 1.84 (0.66) | **0.005** |
| **R^2^ (total fixed effect)** |  | **0.112 (0.06-0.194)** |  | **0.149 (0.09-0.23)** |  | **0.095 (0.055-0.148)** |  |
| **Models with Vascular WM Score as an outcome** | | | | | | | |
| Vascular risk factors | Intercept | 351.65 (40.33) | **<0.001** | 493.86 (83.66) | **<0.001** | 279.45 (7.98) | **<0.001** |
|  | Time | -1.49 (0.38) | **<0.001** | -3.87 (0.47) | **<0.001** | -5.41 (0.44) | **<0.001** |
|  | Age | -3.73 (0.66) | **<0.001** | -5.63 (1.16) | **<0.001** | -2.90 (0.82) | **<0.001** |
|  | Male | - | - | 18.02 (6.46) | **0.006** | 14.96 (6.56) | **0.023** |
|  | CMC | - | - | -8.70 (2.60) | **<0.001** | -5.66 (2.57) | **0.029** |
|  | Smoking | - | - | - | - | -1.27 (6.15) | 0.84 |
|  | CKD | 1.47 (18.79) | 0.94 | - | - | - | - |
|  | Time*CMC | - | - | -0.55 (0.20) | **0.007** | 0.37 (0.16) | **0.021** |
|  | Time*Smoking | - | - | - | - | -1.01 (0.39) | **0.009** |
|  | Time*CKD | -5.71 (2.68) | **0.034** | - | - | - | - |
| **R^2^ (total fixed effect)** |  | **0.112 (0.065-0.189)** |  | **0.201 (0.147-0.275)** |  | **0.109 (0.081-0.182)** |  |
| Gait measures | Intercept | 337.18 (40.0) | **<0.001** | 498.84 (84.93) | **<0.001** | 283.05 (66.05) | 0.80 |
|  | Time | -1.50 (0.38) | **<0.001** | 11.47 (7.11) | 0.11 | -4.90 (0.20) | **<0.001** |
|  | Age | -3.60 (0.66) | **<0.001** | -5.79 (1.18) | **<0.001** | -3.10 (0.81) | **<0.001** |
|  | Male | 12.22 (6.05) | **0.045** | - | - | 13.41 (6.45) | **0.04** |
|  | UPDRS | -3.95 (1.45) | **0.007** | - | - | - | - |
|  | Falls | - | - | -64.05 (21.08) | **0.003** | - | - |
|  | Time*Age | - | - | -0.23 (0.10) | **0.021** | - | - |
| **R^2^(total fixed effect)** |  | **0.148 (0.083-0.24)** |  | **0.168 (0.11-0.24)** |  | **0.097 (0.061-0.145)** |  |
| Behavioral measures | Intercept | 351.65 (40.33) | **<0.001** | 489.72 (86.34) | **<0.001** | 287.10 (64.70) | **<0.001** |
|  | Time | -1.50 (0.38) | **<0.001** | 11.37 (7.12) | 0.11 | -4.90 (0.20) | **<0.001** |
|  | Age | -3.73 (0.70) | **<0.001** | -5.79 (1.19) | **<0.001** | -3.06 (0.80) | **<0.001** |
|  | Male | - | - | 14.31 (6.54) | **0.03** | 13.65 (6.32) | **0.032** |
|  | BAI | - | - | - | - | -2.60 (0.76) | **<0.001** |
|  | Time*Age | - | - | -0.22 (0.10) | **0.022** | - | - |
| **R^2^ (total fixed effect)** |  | **0.112 (0.049-0.172)** |  | **0.156 (0.11-0.22)** |  | **0.129 (0.083-0.196)** |  |
| Plasma markers | Intercept | 413.83 (44.9) | **<0.001** | 462.84 (85.92) | **<0.001** | 303.70 (68.27) | **<0.001** |
|  | Time | -1.50 (0.38) | **<0.001** | 11.42 (7.12) | 0.11 | 1.43 (2.13) | 0.50 |
|  | Age | -3.25 (0.67) | **<0.001** | -4.45 (1.28) | **<0.001** | -2.60 (0.84) | **0.003** |
|  | Male | - | - | 14.16 (6.47) | **0.029** | - | - |
|  | NfL | -21.81 (7.35) | **0.003** | - | - | -11.60 (7.11) | 0.11 |
|  | GFAP | - | - | -22.51 (8.50) | **0.008** | - | - |
|  | Time*Age | - | - | -0.23 (0.10) | **0.022** | - | - |
|  | Time*NfL | - | - | - | - | -1.29 (0.43) | **0.003** |
| **R^2^ (total fixed effect)** |  | **0.142 (0.09-0.24)** |  | **0.178 (0.11-0.24)** |  | **0.10 (0.061-0.172)** |  |
| **Genu-FA**-fractional anisotropy of genu of corpus callosum; **WM**-white matter; **WMH**-white matter hyperintensity; **CMC**- cardiovascular and metabolic condition; **CKD**-chronic kidney disease; **UPDRS**-Unified Parkinson’s disease rating scale; **BAI**-Beck anxiety Inventory, **BDI**- Beck depression inventory; **NfL**-neurofilament light chain; **GFAP**-glial acidic protein | | | | | | | |

**Supplemental Figure 1**: Contributions of individual significant indicators to white matter damage using semi-partial R2 across age groups (low, intermediate (INT), and high-age strata). Genu-FA - fractional anisotropy of genu of the corpus callosum; WM -white matter; WMH - white matter hyperintensity; cardiovascular and metabolic conditions; UPDRS-Unified Parkinson’s disease rating scale; CKD-chronic kidney disease; BMI- Body mass index; BAI-Beck anxiety Inventory.


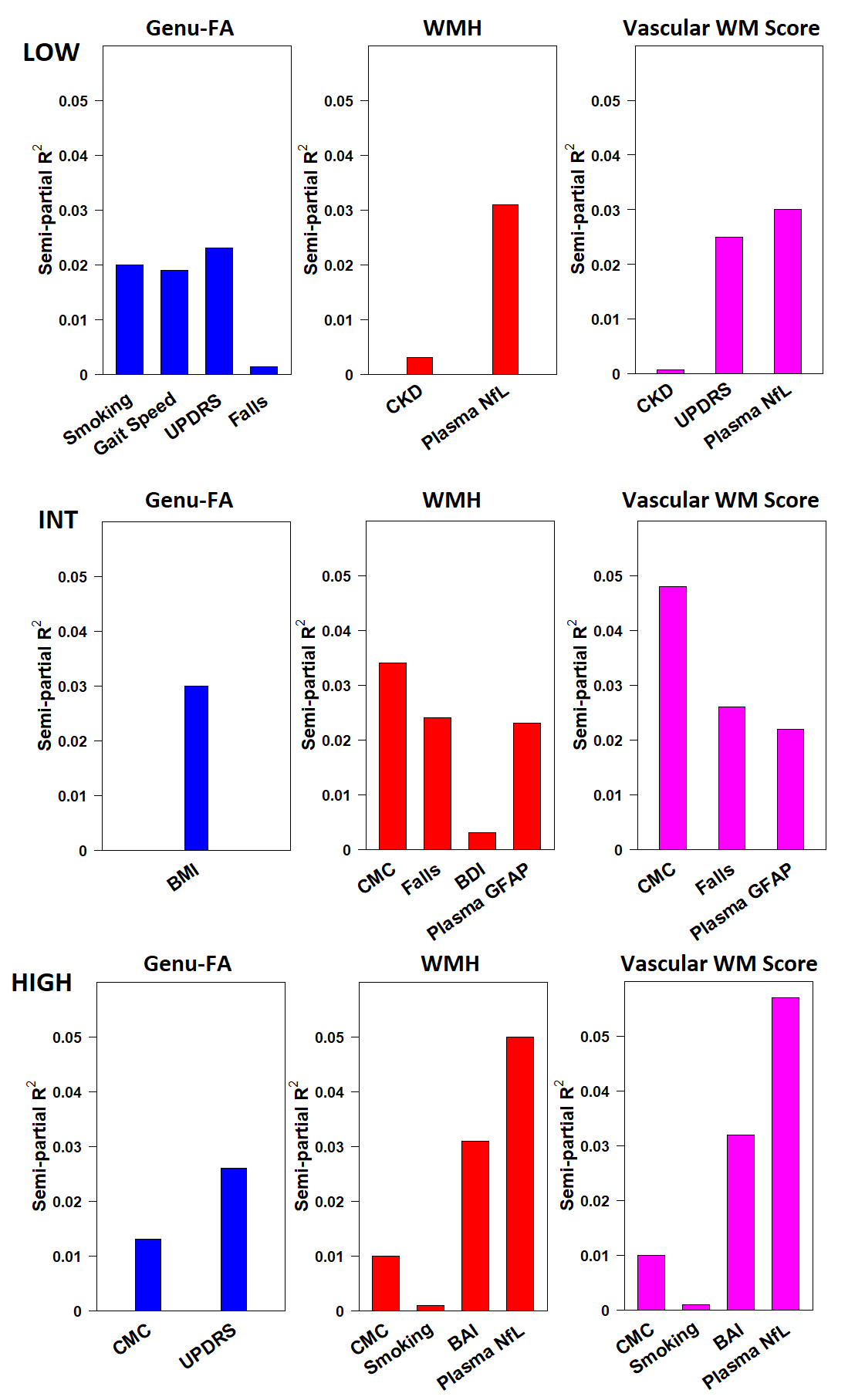


**APPENDIX 1: MRI acquisition and processing**

All participants underwent a 3T head MRI protocol on one of two 3T Siemens Prisma scanners running VE11 software with 64 channel receiver head coils. The protocol included a magnetization prepared rapid gradient echo (MPRAGE) sequence (TR/TE/TI = 2300/3.14/945 ms, flip angle 9°, 0.8 mm isotropic resolution), and a diffusion scan using the product VE11 Simultaneous Multi-Slice acceleration with adaptive coil combination. For the diffusion scan the field of view was 232 mm in X and Y and 162 mm in the Z direction, with 2.0 mm isotropic voxels. The echo and repetition times were 71 and 3400 ms respectively. Data consisted of 127 volumes with 13 non diffusion-weighted images (b=0 s/mm2), and 114 diffusion-encoding gradient directions (6 b = 500, 48 b = 1000, and 60 b = 2000 s/mm2), evenly spread over the entire spherical shells using an electrostatic repulsion model26, and interspersed in time to minimize gradient heating.

The diffusion data were preprocessed using the in-house developed pipeline. After visual inspection, an intracranial mask was made for the diffusion MRI scan1 and the noise in the raw diffusion images was estimated and removed using random matrix theory.2 Then FSL’s eddy_cuda was used to correct for head motion and eddy current distortion,3 followed by the correction of Gibbs ringing4 and Rician bias.5 Diffusion tensors were fitted for both the multi-shell and extracted b=1000 data using a nonlinear least-squares fitting algorithm implemented in dipy,6 from which FA and MD images were generated.

1. Reid RI NZ, Schwarz CG, et al. Diffusion specific segmentation: skull stripping with diffusion MRI data alone. Computational diffusion MRI mathematics and visualization. Cham: Springer; 2018.

2.Veraart J, Novikov DS, Christiaens D, Ades-Aron B, Sijbers J, Fieremans E. Denoising of diffusion MRI using random matrix theory. NeuroImage. Nov 15 2016;142:394-406. doi:10.1016/j.neuroimage.2016.08.016

3. Andersson JLR, Sotiropoulos SN. An integrated approach to correction for off-resonance effects and subject movement in diffusion MR imaging. NeuroImage. Jan 15 2016;125:1063-1078. doi:10.1016/j.neuroimage.2015.10.019

4. Kellner E, Dhital B, Kiselev VG, Reisert M. Gibbs-ringing artifact removal based on local subvoxel-shifts. Magnetic resonance in medicine. Nov 2016;76(5):1574-1581. doi:10.1002/mrm.26054

5. Koay CG, Ozarslan E, Basser PJ. A signal transformational framework for breaking the noise floor and its applications in MRI. Journal of magnetic resonance (San Diego, Calif : 1997). Apr 2009;197(2):108-19. doi:10.1016/j.jmr.2008.11.015

6. Garyfallidis E, Brett M, Amirbekian B, et al. Dipy, a library for the analysis of diffusion MRI data. Frontiers in neuroinformatics. 2014;8:8. doi:10.3389/fninf.2014.00008
